# Assessing Perceived Risk and STI Prevention Behavior: A National Population-Based Study with Special Reference to HPV

**DOI:** 10.1371/journal.pone.0020624

**Published:** 2011-06-02

**Authors:** Amy Leval, Karin Sundström, Alexander Ploner, Lisen Arnheim Dahlström, Catarina Widmark, Pär Sparén

**Affiliations:** 1 Department of Medical Epidemiology and Biostatistics, Karolinska Institutet, Stockholm, Sweden; 2 Department of Learning, Informatics, Management and Ethics, Karolinska Institutet, Stockholm, Sweden; 3 Division of Nursing, Department of Neurobiology, Care Sciences and Society, Karolinska Institutet, Stockholm, Sweden; Tulane University, United States of America

## Abstract

**Introduction:**

To better understand trends in sexually transmitted infection (STI) prevention, specifically low prevalence of condom use with temporary partners, the aim of this study was to examine factors associated with condom use and perceptions of STI risk amongst individuals at risk, with the underlying assumption that STI risk perceptions and STI prevention behaviors are correlated.

**Methods:**

A national population-based survey on human papillomavirus (HPV) and sexual habits of young adults aged 18–30 was conducted in Sweden in 2007, with 1712 men and 8855 women participating. Regression analyses stratified by gender were performed to measure condom use with temporary partners and STI risk perception.

**Results:**

Men's condom use was not associated with STI risk perception while women's was. Awareness of and disease severity perceptions were not associated with either condom use or risk perception though education level correlated with condom use. Women's young age at sexual debut was associated with a higher risk of non-condom use later in life (OR 1.95 95% CI: 1.46–2.60). Women with immigrant mothers were less likely to report seldom/never use of condoms with temporary partners compared to women with Swedish-born mothers (OR 0.53 95% CI: 0.37–0.77). Correlates to STI risk perception differ substantially between sexes. Number of reported temporary partners was the only factor associated for both men and women with condom use and STI risk perception.

**Conclusions:**

Public health interventions advocating condom use with new partners could consider employing tactics besides those which primarily aim to increase knowledge or self-perceived risk if they are to be more effective in STI reduction. Gender-specific prevention strategies could be effective considering the differences found in this study.

## Introduction

From a public health perspective, condom use with temporary partners is a critical primary prevention strategy against sexually transmitted infections (STIs). Their consistent and proper use is the best available means for effectively preventing sexual transmission of viral and bacterial infections ranging from HIV and HPV, an oncogenic virus, to Chlamydia and gonorrhea [1], [2], [3]. Studies on condom use are often limited to specific subgroups such as students, sex workers or abortion seekers. Recent Swedish studies have shown negative trends in condom use and increasing STI prevalence despite well-established programs of public education and easy access to condoms [4], [5].

Public health research and interventions are often based on a knowledge, attitudes and practice (KAP) assumption, which postulate a correlation between knowledge and attitudes and practice or behavior [6], [7], [8], [9], [10]. Knowledge about a risk such as an infection is assumed to effect individuals' attitudes toward potential risk and thus their risk perceptions, which are in turn assumed to effect behavior associated with that risk, such as engagement in prevention strategies. STI prevention strategies often aim to increase knowledge of STIs (**K**nowledge), assuming knowledge will heighten STI risk perception (**A**ttitudes), thereby leading to safer sex practices such as condom use with temporary partners (**P**ractice) [11]. Perceptions of risk and their actual correlations to behaviors need to be further examined to facilitate optimal prevention strategies. Considering condoms' documented effectiveness in disease prevention, their wide-spread availability and public awareness of their purpose in preventing STIs, their non-use in high risk situations such as those with temporary sexual partners warrants further investigation.

In an attempt to understand why people are not engaging in STI prevention practice, a wide array of factors including lack of specific STI knowledge, low risk awareness, low self-efficacy or general risk-taking tendencies amongst certain individuals are often discussed [12], [13]. These factors are in turn believed linked with certain demographic characteristics such as young age or lack of education [14], [15]. Thus the primary aim of this study is to investigate which factors are associated with the practice of condom use with temporary partners, including knowledge and attitudes such as STI risk perception. The secondary aim of this study is to investigate factors associated with STI risk perception due to the hypothesis that risk perception is indicative of engagement in prevention behavior.

## Methods

### Ethics statement

Participation in the study was voluntary, which was explicitly stated in the study invitation letter. By answering the questionnaire, respondents accepted participating in the study and informed consent was given. Separate informed consent forms are not required by the Ethical Review Board for questionnaire studies and were not used here either. Informed consent included register linkages (such as with Statistics Sweden) which was also explicitly stated in the study invitation letter. Ethical approval was granted by the Ethical Review Board of Karolinska Institutet, Solna, Sweden.

### Design and study population

Data is drawn from a national cross-sectional population-based survey conducted in Sweden during January-May 2007 called “Attitudes toward HPV vaccination” [16]. Women (n = 16000) and men (n = 4000) aged 18–30 with a registered address in Sweden were randomly selected from the Swedish Population Register. Response rates were 55% and 43% respectively, with 8855 women and 1712 men participating. More women than men were sampled due to women being the main target of HPV vaccination and to enable power in a future follow-up survey. The sample sizes in this survey for both men and women were calculated to be sufficient to measure proportions with a precision level of 3% and test for differences in proportions at 90% power for a wide range of plausible scenarios.

Invitation to respond to web-based questionnaires was offered to potential participants via a letter. In a first reminder, paper questionnaires were offered to those respondents unable to answer via Internet. If neither had been completed, a telephone reminder was made and the questionnaire answered via a phone interview. The data collection method has previously been described [16], [17]. Using Sweden's unique personal identification numbers survey data was linked with socio-demographic data from Statistics Sweden's database, thus allowing socio-demographic comparison of non-respondents versus respondents [18].

### Operationalization of constructs and outcomes

To test the implicit assumption that risk perceptions are indicative of engagement in prevention practice, knowledge, attitude and practice (KAP) were operationalized as follows: ‘knowledge’ is tested here in questions pertaining to HPV awareness and transmission and cervical cancer familiarity; ‘attitudes’ are captured in responses to STI risk perception as well as in questions pertaining to disease severity perceptions; ‘practice’ is measured via condom use with temporary partners.

Outcome variables investigated in this analysis were condom use with temporary partners in the past year and STI risk perception. Reported condom use with temporary partners was used as an indicator of engagement in a primary disease (STI) prevention strategy. Temporary partners are the focus due to the increased infection risk from serial and or concurrent partners. We chose not to focus on condom use with steady partners as infection risk is conceivably lower in steady relationships with monogamous assumptions, and factors motivating condom use in such relationships could primarily relate to birth control, as opposed to STI prevention behavior.

#### Condom use with temporary partner

Respondents to the question ‘When you had sex with your temporary partner(s), how often did you use a condom during the past year?’ (n = 2594) were included in analysis on condom use with temporary partners. Individuals who reported not having sex with a temporary partner in the past year were excluded, as were non-respondents and respondents with missing data on this question. The remaining respondents were aggregated into the groups: 1) Always/almost always, 2) Often/sometimes, and 3) Seldom/never.

#### STI risk perception

Respondents who reported having had sexual intercourse at any time point and who also answered the question ‘How large a risk do you think you have of contracting an STI?’ (n = 9820) were included in the analysis of STI risk perception. Respondents with no sexual experience, non-respondents and respondents with missing data on this question were excluded. Data were aggregated into three categories for the descriptive analysis: 1) No/small perceived risk, 2) Somewhat large/large perceived risk, and 3) Don't know. Data were further dichotomized into two categories for the regression analysis: 1) No/small perceived risk and 2) Somewhat large/large perceived risk. Those responding ‘Don't know’ were categorized as missing (n = 534) in regression analysis.

Covariates from questionnaire and registers considered in the models included variables on age, education, income, employment type, social welfare status, geographic location, birth country, parent birth country, relationship status, oral and anal sex habits, type of sexual contact, condom use ever and with temporary and steady partners, age at first intercourse, sex partner number for self and compared to others, temporary sex partner number, sex partner gender, knowledge about reasons for and commonness of cervical cancer, knowing cause and severity perception of genital warts, having heard of HPV, predict more unprotected sex if vaccinated, pap smear screening attendance, belief that men/women can be infected with HPV, belief that HPV is sexually transmitted, and willingness to vaccinate against HPV.

### Statistical Analyses

To study potential associations on outcome variables, we first performed chi-square tests in cross-tabulations on knowledge (e.g. having heard of HPV), attitudes (e.g. genital wart severity perception), reported sexual behaviors (e.g. anal sex ever) and socio-demographic data from Statistics Sweden.

Hypotheses for potential variable relationships were carefully considered and directed acyclic graphs (DAGs) were constructed in order to formulate possible relationships and causal pathways, including possible interaction and effect modification [19]. Based on theses DAGs, a series of univariate regression models then assessed the association between various exposures and the outcomes condom use with temporary partners and STI risk perception. Multicollinearity was examined in a correlation matrix for all variables and no serious such was found except for use of condoms with steady and temporary partners and number of sexual partners and number of temporary sexual partners. Variables significantly associated with these outcomes in univariate models were then considered for inclusion in the multivariable multinomial regression models (condom use) and the multivariable logistic regression model (STI risk).

Further selection of explanatory variables for the final models was done by groups of variables pertaining to knowledge, attitudes, behavioral practices and demographics. Variables were retained based primarily on statistical significance, and examining confidence intervals, but subject-matter pertinence of variables to the outcome variables was also considered in the selection process. When constructing the multivariate models, demographic categories were first examined, followed by categories of behavior, then attitudes and finally knowledge. Exposures significant per category were added one at a time to the demographic model and all variables which were excluded were examined separately in a multivariate model to ensure their assumed non-effect held true in various multivariable constellations.

However, significant interaction effects between gender and most explanatory variables were noted. Stratification, typically used to circumvent interaction [20], did not suffice in this case as the model that was predictive for women was not predictive for men. Two separate models were therefore constructed so as to be able to ascertain which variables were predictive for women and men separately in regard to the study outcomes. This also allowed interpretation of a possible gender non-response bias.

A combined STI model adjusted for variables common in both sexes (plus age) was created to generate an odds ratio (OR) for the gender variable. A p-value of less than 0.05 was considered statistically significant in all analyses. Confidence intervals (CIs) are presented at the 95% level. Statistical analyses were performed using SAS version 9.2.

## Results

### Descriptive characteristics

Almost 90% of study participants were born in Sweden (Table 1). Eighty-seven percent of participating women had at least a high school education; this was similar for men (84%). Approximately one-third of respondents were from large urban areas and the remaining from smaller towns and rural areas. Twenty-nine percent of women and 37% of men reported having temporary sexual partners in the past year. Thirty-one percent of women and 36% of men reported two or more sexual partners in this time period (data not shown). Response rates to the survey were lower for individuals with immigrant backgrounds, less than high school education, receiving social welfare and for men (data not shown). Non-response patterns did not differ by gender for the demographic variables for which it was possible to control.

**Table 1:** Distribution of demographic characteristics and response rate of the study sample.

	Women n (%)	Response rate%	Men n (%)	Response rate%
**Age**				
18–21 yrs	2385 (26.9)	56.2	553 (32.3)	46.0
22–24 yrs	1910 (21.5)	53.9	385 (22.4)	42.2
25–28 yrs	2683 (30.3)	55.5	498 (29.0)	40.8
29–31 yrs	1877 (21.2)	55.4	276 (16.1)	41.2
**Birth country**				
Sweden	7851 (88.6)	58.6	1535 (89.6)	45.0
Other Nordic country	78 (0.8)	52.7	14 (0.8)	43.7
Other country	842(9.5)	39.3	141 (8.2)	30.1
**Mother's birth country**				
Sweden	7163 (80.8)	59.4	1407 (82.1)	45.7
Other Nordic country	384 (4.3)	56.3	65(3.8)	35.9
Other country	1308 (14.7)	45.1	240(14.0)	38.0
**Education**				
< High school	2208 (25.2)	49.6	474 (28.0)	39.4
High school or equal	3529 (40.3)	54.4	776 (45.9)	42.1
> High school	3010 (34.4)	63.8	438 (25.9)	50.7
**Social welfare in family**				
No	8112 (91.6)	57.3	1583 (92.4)	44.3
Yes	743 (8.3)	40.3	129 (7.5)	29.9
**Salary (net euro/year)**				
< 10,000	5681 (64.9)	54.1	950 (56.2)	42.3
10,000–20,000-	1725 (19.7)	58.7	266 (15.7)	44.0
>20,000	1341 (15.3)	61.0	472 (27.9)	44.6
**Relationship status**				
Married/registered partner/in a relationship	6333 (71.7)	NA	950 (55.8)	NA
Single	2492 (28.2)		752 (44.1)	
**Employment category**				
Full time	2835 (32.3)	NA	872 (51.1)	NA
Part time	1216 (13.8)		101 (5.9)	
Unemployed	528 (6.0)		114 (6.6)	
Student	3070 (34.9)		540 (31.6)	
Parental leave	685 (7.8)		9 (0.5)	
Disability/other	440 (5.0)		68(3.9)	
**Living area**				
Large city	3028 (34.2)	53.0	568 (33.1)	40.4
Northern Sweden (small city/rural)	1351 (15.2)	57.4	275 (16.0)	46.6
Southern Sweden (small city/rural)	4476 (50.5)	56.4	869 (50.7)	43.3
**Registration method**				
Internet	4032 (45.5)	NA	777 (45.3)	NA
Post	3612 (40.7)		514 (30.0)	
Telephone interview	1211 (13.6)		421 (24.5)	
**Sexual contacts**				
Heterosexual contacts	7573 (91.4)	NA	1463 (95.0)	NA
Homosexual contacts	74 (0.8)		31 (2.0)	
Bisexual contacts	633 (7.6)		46(2.9)	
**Nr. temporary sex partners in past year**				
0	5553 (70.8)	NA	882 (63.1)	NA
1	935 (11.9)		193 (13.8)	
2–4	987 (12.6)		226 (16.1)	
5–9	276 (3.5)		77 (5.5)	
10–14	51 (0.6)		19 (1.3)	
14+	33 (0.4)		0	
**Age at first intercourse**				
< 15	1380 (17.8)	NA	210 (15.6)	NA
15–18	5207 (67.3)		900 (66.9)	
19+	1148 (14.8)		235 (17.4)	

Forty-one percent of men reported always/almost always using condoms with temporary partners, while the corresponding figure among women was 34%. Half the women who responded to the question reported seldom or never using condoms with temporary partners, while 40% of men responded in this manner. Approximately 10% of sexually active respondents reported considering themselves to have a large risk of contracting an STI, with approximately 5% reporting not knowing (Table 2).

**Table 2:** Outcome variables.

	Women n (%)	Men n (%)	p-value*
**Self-perceived risk of contracting STI**			0.1320
No risk or small risk	6832 (82.7)	1277 (83.4)	
Fairly large or large risk	981 (11.8)	160 (10.4)	
Don't know	440 (5.3)	94 (6.1)	
**Condom use with temp partner past yr**			**0.0003**
Always/almost always (100-75%)	711 (33.6)	196 (40.8)	
Often/sometimes (74-25%)	340 (16.0)	91 (18.9)	
Seldom/Never (24-0%)	1063 (50.2)	193 (40.2)	

*P value from chi square test, assessing differnce in responses between men and women.

**Only respondents that reported being sexually active included. For condom variable only those who had reported having a temporary partner in past year were included**.

### Condom use with temporary partners

STI risk perception was correlated to condom use with temporary partners for women but not men. Women were approximately three times as likely to report perceiving a high risk of contracting an STI when they report often/sometimes and seldom/never using condoms compared to those women who report always/almost always using condoms with temporary partners. There were no correlations between condom use and variables related to HPV-related cancer or condyloma awareness, knowledge, or disease severity perception.

Women in families receiving social-welfare were more likely to report seldom/never using condoms with temporary partners (OR = 1.59, CI: 1.02–2.46) (Table 3). Notably, women's own country of birth was non-significant in relation to their reported use of condoms with temporary partners, whereas their mother's country of birth was significantly associated with this outcome. Women with mothers born outside Sweden and other Nordic countries were significantly less likely to report inconsistent condom use with temporary partners (OR = 0.53, CI: 0.37–0.77 in reporting seldom/never use compared to women with Swedish or Nordic-born mothers). Being female and younger than 15 at first intercourse was associated with an almost two-fold odds ratio of reporting seldom/never using condoms with temporary partners (OR = 1.95, CI: 1.46–2.60). For both men and women, number of temporary partners during the past year was associated with increased odds of inconsistent condom use (often/sometimes vs. always/almost always use) (Tables 3 and 4). Women were less likely than men to report seldom/never using condoms as their number of partners increased.

**Table 3:** Correlates of condom use with temporary partners for women.

				Adjusted OR	Adjusted OR	p-value*
				(95% CI)	(95% CI)	
Covariate	Always/almost always	Often/sometimes	Seldom/never	Often/sometimes	Seldom/never	
	n (%)	n (%)	n (%)			
**Relationship status**						**0.0001**
Married/in relationship	289 (31.5)	113 (12.3)	516 (56.2)	0.86 (0.64–1.17)	**1.44 (1.16–1.80)**	
Single	420 (35.3)	226 (19.0)	543 (45.7)	1.0	1.0	
**Education level**						**0.0106**
< High school	91 (27.2)	53 (15.9)	190 (56.9)	**1.71 (1.05–2.79)**	**1.87 (1.30–2.71)**	
High school	395 (31.9)	209 (16.9)	634(51.2)	1.33 (0.95–1.87)	**1.40 (1.08–1.80)**	
>High school	223 (41.4)	78 (14.5)	238 (44.1)	1.0	1.0	
**Salary (net euro/year)**						**0.0103**
< 10000	505 (34.7)	229 (15.7)	721 (49.5)	0.74 (0.33–1.63)	1.16 (0.86–1.56)	
10000–20000	109 (27.2)	74 (18.5)	217 (54.3)	1.0	1.0	
>20000	87 (37.3)	33 (14.2)	113 (48.5)	**1.43 (1.15–1.79)**	1.26 (0.85**–**1.87)	
**Social welfare in family**						**0.0139**
No	661 (34.4)	319 (16.6)	942 (49.0)	1.0	1.0	
Yes	50 (26.0)	21 (10.9)	121 (63.0)	0.74 (0.39–1.43)	**1.59 (1.02–2.46)**	
**Country of mother's birth**						**0.0013**
Sweden	593 (33.1)	282 (15.7)	916 (51.1)	1.0	1.0	
Nordic country	22 (24.4)	14 (15.6)	54 (60.0)	1.54 (0.75–3.17)	1.51 (0.86–2.62)	
Other country	63 (46.3)	23(16.9)	50 (36.8)	1.11 (0.71–1.72)	**0.53 (0.37–0.77)**	
**Age at first intercourse**						**0.0001**
<15	91 (21.8)	58 (13.9)	269 (64.3)	1.26 (0.85–1.87)	**1.95 (1.46–2.60)**	
15–18	497 (36.4)	236 (17.3)	634 (46.4)	1.0	1.0	
19+	100 (42.5)	33 (14.0)	102 (43.4)	0.96 (0.60–1.53)	1.12(0.80–1.58)	
**Nr temp sex partners past yr**						**<.0001**
One	278 (33.3)	73 (8.7)	484 (58.0)	1.0	1.0	
2 to 4	347 (37.0)	177 (18.9)	414 (44.1)	**1.66 (1.17–2.35)**	**0.54 (0.43–0.68)**	
5 to 9	70 (26.7)	66 (25.2)	126 (48.1)	**2.60 (1.62–4.15)**	**0.67 (0.46.0.98)**	
10 to 14	11 (22.0)	13 (26.0)	26 (52.0)	**3.31 (1.34–8.19)**	0.74 (0.33–1.63)	
15+	5 (17.2)	11 (37.9)	13 (44.8)	**4.21 (1.35–13.08)**	0.60 (0.20–1.79)	
**STI risk perception**						**<.0001**
None/small	537 (41.4)	166 (12.8)	595 (45.8)	1.0	1.0	
Rather big/large	130 (19.6)	149 (22.5)	383 (57.8)	**2.90 (2.1–4.00)**	**3.03 (2.33–3.92)**	
Don't know	42 (28.4)	24 (16.2)	82 (55.4)	**2.26 (1.27–4.02)**	**2.09 (1.33–3.26)**	
**Anal sex ever**						**0.0055**
Yes	264 (28.3)	152 (16.3)	518 (55.4)	1.16 (0.86–1.56)	**1.43 (1.15–1.79)**	
No	421 (37.9)	178 (16.0)	512 (46.0)	1.0	1.0	
**Sexual contacts**						0.1516
Heterosexual	624 (34.4)	301 (16.6)	891 (49.0)	1.0	1.0	
Bisexual	78 (28.6)	35 (12.8)	160 (58.6)	0.74 (0.46–1.17)	1.25 (0.90–1.72)	
Homosexual	6 (28.6)	4 (19.0)	11 (52.4)	1.11 (0.24–5.05)	0.81 (0.24–2.73)	

*based on likelihood ratio test.

**Descriptive statistics and mutually adjusted multinomial, multivariable regression model. Model adjusted for all variables presented in the table. Always/almost always condom use is reference group. Odds ratios show excess risk of using condoms often/sometimes or seldom/never instead of always/almost always, compared to the reference level of each exposure.**

**Table 4:** Correlates of condom use with temporary partners for men.

				Adjusted OR	Adjusted OR	p-value*
				(95% CI)	(95% CI)	
Covariate	Always/almost always	Often/sometimes	Seldom/never	Often/sometimes	Seldom/never	
	n%	n%	n%			
**Education level**						**0.0058**
< High school	26 (36.1)	10 (13.9)	36 (50.0)	1.03 (0.40–2.61)	**3.41 (1.58–7.35)**	
High school	119 (39.4)	54 (17.9)	129 (42.7)	0.95 (0.52–1.74)	**2.55 (1.44–4.53)**	
>High school	51 (48.6)	27 (25.7)	27 (25.7)	1.0	1.0	
**Salary (net euro/year)**						0.0554
< 10000	138 (46.1)	50 (16.7)	111 (37.1)	0.59 (0.50–2.78)	0.68 (0.74–3.26)	
10000–20000	29 (34.5)	19 (22.6)	36 (42.9)	1.0	1.0	
>20000	28 (30.1)	22 (23.6)	43 (46.2)	1.18 (0.29–1.19)	1.56 (0.37–1.24)	
**Nr temp sex partners past yr**						**0.0002**
One	77 (43.0)	15 (8.4)	87 (48.6)	1.0	1.0	
2 to 4	89 (42.0)	47 (22.1)	76 (35.8)	**2.83 (1.40–5.71)**	0.67 (0.42–1.08)	
5 to 9	27 (37.0)	23 (31.5)	23 (31.5)	**4.40 (1.92–10.05)**	0.62 (0.31–1.23)	
10 to 14	3 (18.7)	6 (37.5)	7 (43.7)	**10.23 (2.21–47.35**)	1.57 (0.37–6.62)	
**Anal sex ever**						**0.0009**
Yes	67 (33.7)	36 (18.1)	96 (48.2)	0.87 (0.49–1.55)	**2.14 (1.35–3.39)**	
No	115 (44.7)	53 (20.6)	89 (34.6)	1.0	1.0	
**Sexual contacts**						0.1531
Heterosexual	182 (40.7)	82 (18.3)	183 (40.9)	1.0	1.0	
Bisexual	6 (31.5)	6 (31.5)	7 (36.8)	2.14 (0.63**–**7.31)	0.83 (0.25**–**2.72)	
Homosexual	8 (57.1)	3 (21.4)	3 (21.4)	0.69 (0.15**–**3.06)	**0.22 (0.05–0.91)**	

*based on likelihood ratio test.

**Descriptive statistics and mutually adjusted multinomial, multivariable regression model. Model adjusted for all variables presented in the table. Always/almost always condom use is reference group. Odds ratios show excess risk of using condoms often/sometimes or seldom/never instead of always/almost always, compared to the reference level of each exposure.**

Women's relationship status was correlated with condom use with temporary partners whereas men's was not (Tables 3 and 4). If married or in a relationship, women were over 40% more likely to report seldom/never using condoms with temporary partners than was the case if single (OR = 1.44, CI: 1.16–1.80) (Table 3). Inconsistent and non-use of condoms were more commonly reported in both women and men with lower education levels (Tables 3 and 4). The data indicated that age had a negative relationship to consistent condom use and education had a positive relationship to condom use (data not shown). As age and education correlate we kept only education in the model, as its effect was stronger in terms of statistical significance and ORs.

Both men and women who reported ever engaging in anal sex were more likely to report seldom/never using condoms with temporary partners (OR = 2.14, CI: 1.35–3.39 for men; OR = 1.43, CI: 1.15–1.79 for women).

Variables on oral sex habits, condom use with steady partner in the past year, condom use ever, pap smear screening attendance, knowing the cause of cervical cancer and willingness to vaccinate against HPV if it was cost-free were significant in the univariate analyses for women but became non-significant in the final models for condom use (data not shown).

### STI risk perception

Although women and men had similar distributions of risk perception (Table 2), there were gender-differences in factors that correlated to risk perception (Tables 5 and 6). Women were almost twice as likely to report perceiving themselves to have large STI risk compared to men (OR = 1.91, CI:1.54–2.3) after adjusting for number of temporary partners, relationship status, and age (data not shown). Relationship status and number of temporary partners were the only correlates common to both women's and men's perceptions of STI risk (Tables 5 and 6). Persons not in a relationship were more likely to report perceiving themselves at high STI risk. Increased self- perception of STI risk was correlated to higher numbers of temporary partners. As noted above, women's but not men's STI risk perception was associated with condom use with temporary partners. Women who reported seldom/never or often/sometimes using condoms with temporary partners were three times more likely to perceive themselves at high STI risk than those reporting use always/almost always (Table 5).

**Table 5:** Correlates to women's STI risk perception.

	Low risk	High risk	Adjusted OR	p-value*
	n (%)	n (%)	(95% CI)	
Covariate			High risk	
**Relationship status**				**<.0001**
				
Married/in relationship	5546 (93.4)	393 (6.6)	**0.39 (0.32–0.47)**	
Single	1271 (68.5)	584 (31.5)	1.0	
**Age**				**<.0001**
18–21	1532 (81.7)	343 (18.3)	**1.58 (1.18–2.12)**	
22–24	1435 (83.5)	283 (16.5)	**2.03(1.52–2.70)**	
25–28	2224(89.9))	251 (10.1)	**1.38(1.03–1.83)**	
29–31	1641 (94.0)	104 (6.0)	1.0	
**Nr temp sex partners past yr** **				**<.0001**
Zero	5107 (95.8)	222 (4.2)	1.03 (0.72–1.48)	
One	693 (81.3)	159 (18.7)	1.0	
2 to 4	594 (64.9)	321 (35.0)	**1.53 (1.17–1.99)**	
5 to 9	112 (42.7)	150 (57.2)	**2.42 (1.56–3.76)**	
10+	28 (33.7)	55 (66.3)	**3.74 (1.84–7.61)**	
**Condom use temp partner past year**				**<.0001**
Always/almost always	537 (80.5)	130 (19.5)	**1.0**	
Often/sometimes	166 (52.7)	149(47.3)	3.06 (2.22–4.23)	
Seldom/never	595 (60.8)	383 (39.2)	3.11 (2.41–4.03)	
**Nr sex partners past yr**				**<.0001**
Zero	199 (89.2)	24 (10.8)	1.62 (0.94–2.8)	
One	4828 (96.3)	185 (3.7)	1.0	
2 to 4	1177 (75.7)	378 (24.3)	**2.43 (1.82–3.26)**	
5 to 9	302 (59.0)	210 (41.0)	**2.78 (1.89–4.09)**	
10 to 14	61 (49.6)	62 (50.4)	**3.51(1.98–6.21)**	
15+	63 (50.4)	62 (49.6)	**2.92(1.56–5.45)**	
**Compare number of sex partners with other people**				**<.0001**
More	633 (69.6)	276 (30.3)	**2.262 (1.76–2.79)**	
Less	3216 (93.2)	233 (6.8)	**0.70 (0.57–0.87)**	
Same	2286 (85.8)	379 (14.2)	1.0	
Don't know	477 (90.0)	53 (10.0)	0.81 (0.54–1.20)	

Low risk is defined as responses no or low risk for contracting STIs and high risk defined as responses rather high or high risk for contracting STIs.

*Based on likelihood ratio test.

**Odds ratios here describe probability of high self-perceived risk for contracting an STI assuming always/almost always use of condoms with temporary partners.

**Descriptive statistics and mutually adjusted binomial, multivariable regression model. Model adjusted for all variables presented in the table. Probability modeled low STI risk.**

**Table 6:** Correlates to men's STI risk perception.

	Low risk	High risk	Adjusted OR	p-value*
	n (%)	n (%)	(95% CI)	
Covariate			High risk	
**Sexual contacts**				**0.0012**
Heterosexual	1223 (89.9)	137 (10.1)	1.0	
Bisexual	32 (69.6)	14 (30.4)	**3.84 (1.77–8.30)**	
Homosexual	20 (69.0)	9 (31.0)	2.33 (0.83–6.40)	
**Anal sex ever**				**0.0016**
Yes	478 (84.3)	89 (15.7)	**1.99 (1.29.3.06)**	
No	736 (92.2)	62 (7.8)	1.0	
**Relationship status**				**0.0127**
Married/in relationship	832 (93.9)	54 (6.0)	**0.54 (0.33–0.87)**	
Single	440 (80.7)	105 (19.3)	1.0	
**Nr temp sex partners past yr**				**<.0001**
Zero	821 (96.7)	28 (3.3)	**0.27 (0.14–0.51)**	
One	151 (84.8)	27 (15.2)	1.0	
2 to 4	165 (79.3)	43 (20.7)	1.55 (0.88–2.74)	
5 to 9	47 (63.5)	27 (36.5)	**2.79 (1.43–5.44)**	
10 to 14	10 (55.6)	8 (44.4)	**3.41 (1.13–10.23)**	

**Descriptive statistics and mutually adjusted binomial, multivariable regression model. Model adjusted for all variables presented in the table. Probability modeled low STI risk.**

Low risk is defined as responses no or low risk for contracting STIs and high risk defined as responses rather high or high risk for contracting STIs.

*Based on likelihood ratio test.

Condom use with steady partners was not associated with STI risk perception, whereas use with temporary partners was for women. Women reporting having had more sex partners than other people (versus less or same number), were twice as likely to perceive large STI risk. Men's STI risk perception was correlated with anal sex whereas women's was not (Tables 5 and 6). Men who reported ever having had anal sex were twice as likely to report large STI risk (Table 6). This was controlled for bisexual contacts which were a significant risk factor for men but not for women in terms of high self-perceived STI risk. Men reporting bisexual contacts had an odds ratio of 3.84 to consider themselves at high STI risk, compared to men reporting only heterosexual contacts, while men reporting homosexual contacts had a non-significant odds ratio of 2.33.

Variables related to HPV-related cancer or condyloma awareness, knowledge or disease severity perception were non-significant in the univariate and multivariable models for STI risk perception (data not shown).

## Discussion

This population-based study revealed that condom use with temporary partners was not associated with STI risk perception for men whereas it was for women, despite a higher percentage of men reporting consistently having used condoms with temporary partners than women. It is particularly notable that correlates to STI risk perception differ substantially between men and women. Awareness and severity perceptions of HPV and HPV-related cancer were not associated with either condom use or risk perception, whereas education level was positively associated with condom use. Women who were youngest at sexual debut also had two-fold increased odds of reporting non-condom use with temporary partners compared to women with later sexual debuts. Also, women with immigrant mothers were almost twice as likely to report using condoms consistently with temporary partners compared to women with Swedish-born mothers. Number of reported temporary partners was the only common factor associated for both men and women with condom use and STI risk perception.

Based on an underlying KAP assumption, we expected to find those with higher levels of HPV awareness or disease severity perceptions also reporting more consistent condom use with temporary partners. The fact that these variables were not at all associated was surprising, as was the finding that they were not associated with STI risk perception either. This may point to an ineffectiveness of KAP assumptions in explaining this area of risk and prevention practice. This also points to education level's correlation to condom use as an effect of socio-economic status rather than an effect of disease knowledge. High socio-economic status often reduces barriers to prevention for both chronic and infectious diseases [21].

Our findings regarding men's STI risk perception and condom use correlates are particularly disconcerting considering the prioritized and liberal views of sexual education in Swedish schools [22]. The Health Belief Model (HBM) is similar to KAP in that it linearly associates perceived threat of a disease with the likelihood of taking preventive health action [8]. Even though KAP and HBM assumptions are supported by public education policy on sexual health, they do not appear predictive of the practice of condom use in both sexes. This calls for a rethinking of educational and public policy in efforts to promote condom use to reduce STI burden. This study reveals that efforts primarily aimed to increase STI awareness and/or perceptions of risk will not suffice in influencing prevention behavior. A deeper understanding into the actual barriers individuals experience in engaging in prevention behavior, with subsequent strategizing to alleviate these barriers, is necessary in the sphere of public health epidemiology.

In societies where health risk exposure information is abundant, as is the case today in countries with high GDP per capita such as Sweden or the U.S., few epidemiological studies aim to measure how individuals interpret their risk exposure and whether or how this is in turn associated with prevention behavior. Our study shows vast gender differences in how risk is perceived and correlated to prevention behavior. Future attempts to measure possible causes and effects of risk perceptions should also aim to measure gender differences.

Other studies have indicated differences in prevalence of male and female condom use but no other large-scale population-based studies using similar variables have evaluated men and women with separate models [15], [23], [24], [25]. The measure of condom use itself is often dubious in terms of partner type, frequency and duration of use and analysis of data is often descriptive only or, if analytic, adjusted only for a limited number of factors [5], [26], [27], [28]. It is possible that condom use is primarily associated with pregnancy, and not STI, prevention [25]. Lazarus et al. noted high oral contraception use amongst Swedish women, which could partially explain the lower condom use among women found in our study [29]. They also noted that low HIV prevalence in Sweden could explain low condom use; however our study shows that men do not correlate condom use to STI risk, so our data does not necessarily support this theory. However, the association of heterosexual men reporting anal sex perceiving themselves to be at higher STI risk perhaps indicates that men do not find vaginal sex as ‘risky’ due to a belief that sexual risk means HIV which is seen as a homosexual and hence anal sex, risk. This could be an example of how risk education messages are internalized in a manner different from those who broadcast them intended.

It should be recognized however that as data were collected cross-sectionally we cannot make any inference about cause and effect. This makes it difficult to interpret the significance shown here of reported relationship status in the models as concurrency is unknown. Another potential limitation to our study is respondents self-define ‘temporary’ when asked about condom use with temporary partners in the past year. To avoid recall bias we limited our questions to only asking about sexual relationships in the past year.

Women with mothers born outside the Nordic countries reported more frequent condom use with temporary partners than those with Swedish or Nordic-born mothers. We have not found adequate explanation in the literature for this unexpected finding. One interpretation might be that these women are raised with different values and hence develop different practices than their peers, but it could also be due to differences in reported versus practiced behavior. In light of our findings, maternal and cultural influences on STI prevention behavior merit further investigation.

Specific to HPV, early sexual debut is a well-known risk factor for developing cervical cancer. This is thought to be due to exposing the cervical transformation zone to HPV infection for a longer time-period and/or an average increased number of lifetime sexual partners [30], [31], [32]. We found that young sexual debut age correlated to a nearly two-fold odds ratio for non-condom use with temporary partners, which highlights that other risk-related behaviors than age at sexual debut may contribute to the risk of developing cervical cancer. Factors interacting with age at first intercourse on cervical cancer risk warrant further investigation.

Other studies have shown low condom use to be related to a wide variety of factors, including decreased sensation, partner disapproval, non-communication, low levels of emotional intimacy and alcohol use [15], [33], [34], [35]. Höglund et. al's study suggests that attitudes to using condoms with new partners were overwhelmingly positive among high school students, though again our study suggests that these attitudes do not necessarily translate into actual behavior [36].

This study's population-based sampling frame enhances its generalizability in Sweden and also its relevance in other contexts with similar demographics and social climates. The majority of studies on STI risk and condom use rely on convenience-sampling amongst a selected group, e.g. university students or sex workers. To our knowledge, few national population-based studies of this nature have been conducted outside the Nordic region. With consideration given to the sensitive character of the questions and healthy young population targeted, our ∼50% participation rate can be seen as acceptable [37]. We did have a slight underrepresentation of men, immigrants, those receiving social welfare, and those with lower education (Table 1), but we found no gender-specific interaction effects amongst non-respondents. There is no indication that only a specific group responded, augmenting the study's generalizability to young Swedish adults.

Because the survey was based on a random selection of the population, this minimizes the problem of selection bias. However there is always the potential for a non-response bias, in which those who chose not to participate deviated in regard to the outcome variables under investigation. The possibility of non-response bias in the sexual habits questions cannot be ruled out completely, although the distribution of sexual habits and number of survey respondents whom had not made their sexual debut appeared to be reasonable, reflecting the relative heterogeneity expected in the population. Furthermore, both men and women proportionally indicated similar risk perception levels (Table 2), so no non-response bias based on gender specifically can be ascertained with that outcome variable. The proportionality of condom use responses did differ based on gender, with men more apt to report consistent use. Therefore, we cannot rule out the potential for non-response bias for that variable. However, as seen in Table 2 there is still a clear within-gender response variation for condom use, minimizing the likelihood that non-response bias would be particularly problematic based on this outcome.

Having had multiple sexual partners, or having a partner who has had multiple sexual partners, puts one at risk for a variety of infections such as HIV, Chlamydia, HPV and gonorrhea [38]. Even with treatments available today in high resource countries against many of these diseases, contracting one can lead to a plethora of undesirable effects such as significant loss in quality of life, social stigma, antibiotic resistance, impaired fertility and preterm births [22]. Early death is related not only to AIDS and cervical cancer but also to other HPV-related cancers – e.g. penile, anal, oral and oropharyngeal malignancies. With these detrimental health effects, proper condom use as a primary prevention measure should remain a top priority for health officials [4], [22], [39]. This study concludes however, that campaigns with a primary aim to increase STI knowledge and awareness with the intention of influencing risk perceptions amongst those sexually active, may not effectively translate into an increase in prevention behaviors. To reach the public health goal of reducing STI prevalence, barriers to engaging in STI prevention need to be addressed, including gender barriers as this study highlights.
